# pH trends and seasonal cycle in the coastal Balearic Sea reconstructed through machine learning

**DOI:** 10.1038/s41598-022-17253-5

**Published:** 2022-07-28

**Authors:** Susana Flecha, Àlex Giménez-Romero, Joaquín Tintoré, Fiz F. Pérez, Eva Alou-Font, Manuel A. Matías, Iris E. Hendriks

**Affiliations:** 1 Instituto de Ciencias Marinas de Andalucía (ICMAN-CSIC), Polígono Río San Pedro s/n, 11519 Cádiz, Puerto Real Spain; 2 Instituto Mediterráneo de Estudios Avanzados (IMEDEA-UIB-CSIC), Miquel Marquès 21, 07190 Esporles, Spain; 3 Instituto de Física Interdisciplinar y Sistemas Complejos, (IFISC-UIB-CSIC), Campus UIB, 07122 Palma, Spain; 4 Balearic Islands Coastal Observing and Forecasting System (SOCIB), Parc Bit, Naorte, Bloc A 2op. pta. 3, 07121 Palma, Spain; 5 Instituto de Investigaciones Marinas (IIM-CSIC), Eduardo Cabello 6, 36208 Vigo, Spain

**Keywords:** Carbon cycle, Marine chemistry, Marine chemistry

## Abstract

The decreasing seawater pH trend associated with increasing atmospheric carbon dioxide levels is an issue of concern due to possible negative consequences for marine organisms, especially calcifiers. Globally, coastal areas represent important transitional land-ocean zones with complex interactions between biological, physical and chemical processes. Here, we evaluated the pH variability at two sites in the coastal area of the Balearic Sea (Western Mediterranean). High resolution pH data along with temperature, salinity, and also dissolved oxygen were obtained with autonomous sensors from 2018 to 2021 in order to determine the temporal pH variability and the principal drivers involved. By using environmental datasets of temperature, salinity and dissolved oxygen, Recurrent Neural Networks were trained to predict pH and fill data gaps. Longer environmental time series (2012–2021) were used to obtain the pH trend using reconstructed data. The best predictions show a rate of $-\,0.0020\pm 0.00054$ pH units year$^{-1}$, which is in good agreement with other observations of pH rates in coastal areas. The methodology presented here opens the possibility to obtain pH trends when only limited pH observations are available, if other variables are accessible. Potentially, this could be a way to reliably fill the unavoidable gaps present in time series data provided by sensors.

## Introduction

Atmospheric carbon dioxide (CO$_{2}$) emissions are exponentially increasing since the industrial revolution, principally due to fossil fuel use, industry and land-use change. Around a 46% of this CO$_{2}$ remains in the atmosphere while the rest is captured by natural compartments: the terrestrial biosphere and the ocean^1^. At present, the oceans have absorbed around an estimated 26% of the total anthropogenic CO$_{2}$ released from 2011 to 2020^1^. Once CO$_{2}$ dissolves in seawater, a sequence of chemical reactions occurs that derives in an increase of [H$^{+}$] ions, which results in a decrease in seawater pH. This process, a consequence of increasing atmospheric CO$_{2}$, is termed Ocean Acidification (OA)^2^. In addition to the pH decrease, [H$^{+}$] ions react with carbonate ions [CO$_{3}^{2-}$] to form [HCO$_{3}^{-}$], leading to a reduction of the [CO$_{3}^{2-}$] ion levels^3^. Low carbonate levels affect the saturation state of calcium carbonate minerals, increasing difficulties in shell-forming for calcifying marine organisms (e.g., plankton, mollusks, echinoderms and corals). Consequences of OA are an important threat to marine ecosystems visible in higher levels of the trophic chain, with complex and wide-ranging impacts on the physiology of different species and therefore on their metabolism^4,5^. These metabolic effects will have numerous consequences at an organism scale, in particular, they can cause a decrease in growth, locomotion, reproductive capacity and homeostasis if they are not capable to control the conditions for calcification^6^. Negative effects of this magnitude could cause an unexpected cascade effect impacting on the structure and functions of ecosystems and trophic networks^7^ and cannot be easily generalized.

Also, ocean CO$_{2}$ uptake and derived OA are not homogeneous at the global scale, with some areas more affected. For instance, the Mediterranean Basin is an area where effects are stronger compared to the global ocean^8^. The Mediterranean Sea, constituting only a 0.82% of the surface and 0.32% of the volume of the global ocean, is cataloged as one of the most complex marine ecosystems, defined as a “miniature ocean”^9^, inhabited by an extensive and diverse biota that represents between 4 and 18 % of the world’s total marine species^10^ and serves as a model^9^ to anticipate the responses of the global ocean to different types of pressures. It has been also defined as a climate change “hot spot”^8^, whit OA and its derived consequences characterized as one of the climatic threats with the greatest potential impact, followed by the temperature and UV radiation increase^11^. The temperature rise in this semi-enclosed sea is expected to be two to four-fold times higher than that in the global ocean^12,13^. In addition, the sixth assessment report (AR6) of the IPPC7, places a high level of confidence on the increase in frequency of heatwaves and ongoing ocean acidification^14^. Recent studies have confirmed that there is a trend of around 0.34 $^{\circ }$C warming per decade in the Mediterranean Outflow Water (MOW) through the Strait of Gibraltar towards the Atlantic Ocean^15^, associated with decreasing values of pH. Furthermore, in the Mediterranean Sea, due to its biogeochemical and hydrodynamic characteristics, such as the high alkalinity of its waters and the active thermohaline circulation^16^, there is a larger absorption of atmospheric CO$_{2}$ and an intense transport of this CO$_{2}$ from the oceanic surface to deep areas^17,18^, already observed in the MOW^19,20^, with estimated OA trends of − 0.0044 pH units per year in the Strait of Gibraltar^19^ and ranging from − 0.0017 to − 0.003 in the Mediterranean Basin^21,22^.

The Mediterranean Sea has an extensive coastline, which extends for 46,000 km and is shared by 21 countries^23^. Coastal zones, as transitional areas, are inherently complex systems due to the strong biogeochemical-physical coupling, occurring relevant biogeochemical exchanges. Interactions in coastal areas involve terrestrial inputs of nutrients and particulate matter from river runoff and groundwater discharges, oceanic forcing (waves, tides, and currents), and atmospheric exchange of aerosols and trace gases, all of them which are influenced by the intense human activity in the coastline^24^. Hence, processes related to the carbon system in coastal areas are more dynamic and complex than in the open ocean^25^, and the range of pH change between − 0.023 and 0.023 pH units per year^26^ is therefore $\sim \,35$ times larger than in the open ocean with − 0.0013 to − 0.0026 pH units year$^{-1}$^27^. In particular, anthropogenic CO$_2$ inputs appear to play a minor role compared to other sources of variability in coastal zones^28^.Therefore, it is difficult to foresee how the pH conditions in the coastal areas in the year 2100 will differ from the present, due to the lack of knowledge on precise current pH values in the different coastal ecosystems and their variability obtained from long time series. Carbonate chemistry and in particular pH fluctuations are characterized by a wide spatial heterogeneity and temporal variability (daily and seasonal oscillations) in coastal ecosystems^28–30^. The variability of pH is determined by a wide range of physical and biogeochemical processes, from mesoscale hydrological processes to small-scale metabolic processes^31^.

The primary production in the western Mediterranean Sea is characterized by a seasonal variability induced by the increase of the surface layer nutrients by the winter vertical mixing in the water column^32^. In addition, the presence of macrophytes^33^in the coastal areas of the northern Mediterranean Sea, mainly the endemic *Posidonia oceanica* whose meadows extend from the surface to 30–40 m depth, are defined as highly productive habitats. In these ecosystems, variability tends to follow daily and seasonal cycles, since biological metabolism is responsible for variations in the concentrations of oxygen (O$_{2}$) and CO$_{2}$^29,34^. Thus, increasing pH values are expected for autotrophic ecosystems (production > respiration) during daylight hours. Indeed, recent studies indicate that seagrass meadows can locally alleviate low pH conditions for extended periods of time with important implications for the conservation and management of coastal ecosystems^35^.

Nevertheless, changes in pH can appear idiosyncratic and display a diversity of patterns depending on the coastal area under consideration, as many drivers of the carbon system can influence these variable ecosystems, including temperature variability, biological activity and terrestrial and open ocean inputs^26^. Therefore, the properties of the carbon system have to be evaluated while taking into account the different interactions in every area. To the present day, there is still a lack of understanding of how coastal areas behave and how they contribute to the global carbon budget, also in part due to the intensive effort necessary to obtain representative time series of the carbon system data according to standard practices. The Global Ocean Acidification Observing Network (GOA-ON) defined that the accuracy included in the “weather goal” should be better than 0.02 and for the “climate goal” < 0.003 pH units^36^. Instrumentation for autonomous pH measurements have improved in recent years and production costs have come down, but they remain complex and relatively expensive. For climate change studies commercial oceanographic instrumentation barely accomplish the GOA-ON “climate goal” accuracy recommendation, with only spectrophotometric devices and Ion Sensitive Field-Effect Transistors (ISFETs) based pH probes reaching the standards. In this sense, the SAMI-pH sensor (Submersible Autonomous Moored Instrument, Sunburst Sensors, LCC), based on spectrophotometric techniques has been denoted as an excellent pH sensor for OA studies^19^. However, the maintenance of oceanographic time series stations entails several operational and non-operational difficulties, involving financial costs, meteorological risks (i.e. bad conditions for navigation, instrumentation loss, etc.), deployment in areas with high transit, issues essentially related to the sensor itself (i.e. instrumental failure) and possible human errors. Therefore, the appearance of data gaps is common, implying the lack of pH data obtained using high-quality instrumentation for global carbon studies.

Currently, novel computational methods based on Machine Learning (ML) are allowing to tackle these data absence difficulties. Machine Learning is a part of Artificial Intelligence that has attained a mature status in the last decade or so, particularly through the so-called Deep Learning (DL)^37^, with major advances in solving problems that have resisted the best attempts of the artificial intelligence community for many years^38^. In particular, some DL techniques are useful in time series forecasting^39^ and also in the reconstruction of coupled time series^40^, such as Recurrent Neural Network (RNN) architectures like Long Short-Term Memory (LSTM)^41^ or Gated Recurrent Unit (GRU).

Nowadays, there is an increasing number of studies that use DL to understand the processes involved in the carbon system variability, but mainly focused on the open ocean^42–46^, while relatively few studies focused on coastal seas^47,48^ and none specifically in the Mediterranean coastal Sea, perhaps because of the complexity and heterogeneity of the basin and its continental shelves. Therefore, the main objective of this study is to obtain the trend for pH decrease in the coastal Balearic Sea by applying Machine Learning techniques. In addition, this study aims to provide a useful tool to fill gaps in pH time series and to reconstruct pH data when additional environmental variables are available.

**Figure 1:**
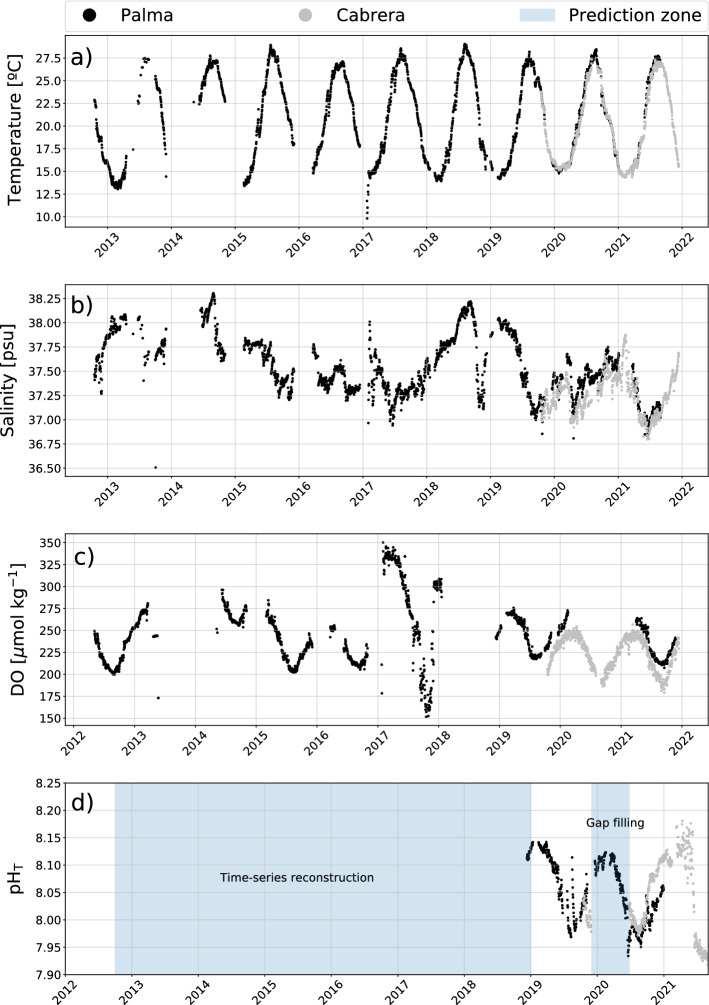
Daily averaged time series data from the Bay of Palma (black dots) and Cabrera stations (grey dots): (**a**) temperature ($^{\circ }$C), (**b**) salinity (psu), (**c**) dissolved oxygen (DO) (${\upmu \text {mol kg}^{-1}}$) and (**d**) pH$_{T}$ in pH units. The pH time series of the Bay of Palma will be reconstructed in the period 2012–2021 while only gaps will be filled in Cabrera, as marked in blue in the figure.

## Results

### Time series data

The collection of pH values, in total scale (pH$_{T}$), started in December 2018 in the Bay of Palma, recording data almost continuously until the end of 2021. In the Cabrera station, pH$_{T}$ was obtained from November 2019 to December 2021, with a relevant data gap from December 2019 to June 2020 (Fig. 1d) due to a sensor malfunction with a reparation prolonged for an extended period of time owing to the Covid-19 lockdown. Additional environmental parameters like temperature, salinity and dissolved oxygen (DO) concentration are available from the Bay of Palma station since 2012, while only a limited time series of these variables (since 2019) is available for Cabrera (Fig. 1a–c).

In both stations, temperature ranged from a minimum of 12.99 $^{\circ }$C to maximum values of 29.07 $^{\circ }$C from 2012 to 2021, with no observed differences between the stations in Cabrera and the Bay of Palma (Fig. 1a). The surface water temperatures are a clear representation of the typical Mediterranean climate seasonality with mild winters and warm to hot summers. Salinity did not show a repetitive seasonal pattern between years in either stations. However, in Cabrera salinity is slightly lower than in the Bay of Palma. During the data acquisition period, the lowest salinity value of 36.83 was found in Cabrera and highest of 38.30 in the Bay of Palma (Fig. 1b).

The surface water of the coastal sites in the Balearic Sea in the Palma Bay and the Cabrera stations was highly saturated with oxygen during all the seasons, with DO concentrations up to 348.94 ${\upmu \text {mol kg}^{-1}}$ during winter and of 150.66 ${\upmu \text {mol kg}^{-1}}$ during the summer and early autumn (Fig. 1c). pH$_{T}$ values obtained starting in December, 2018 to December, 2021 increased during winter reaching up to 8.18 pH units at in situ temperature and decreasing to 7.91 pH units in summer, with the highest variability and maximum and minimum values measured in Cabrera (Fig. 1d).

Considering sampling period of the additional (temperature and salinity) and calculated parameters (Total Alkalinity; TA) was larger in the Bay of Palma compared to Cabrera, we evaluate the linear tendencies with time for the Bay of Palma variables. The sea surface temperature in the Bay of Palma increased with a rate of 0.035 ± 0.008 $^{\circ }$C per year (R$^{2}$ = 0.008, *p*-value $<$ 0.001) from 2012 to 2021, whereas the salinity decreased significantly with − 0.059 ± 0.002 psu per year (R$^{2} = 0.25$, *p*-value < 0.001). The annual trend for TA, clearly related to the decrease in surface salinity, showed a relevant decrease of − 4.0 ± 0.4 ${\upmu \text {mol kg}^{-1}}$ (R$^{2} = 0.0379$, *p*-value < 0.001), supported by the discrete water samples for TA obtained during the period from 2019 to 2021 (Fig.  S3).

### Reconstruction pH time series with deep learning

The amount of available pH$_{T}$ data from both Palma Bay and Cabrera stations is comparable and relatively short (mostly in Cabrera), but the length of the additional ambient data (temperature, salinity and DO) differs enormously among stations. Thus, there is a need to approach the time series prediction problem for both sites with different objectives. Common to both sites, a DL model with a RNN architecture will be developed to predict the pH$_{T}$ time series from the accompanying ambient data (temperature, salinity and dissolved oxygen), which are expected to be correlated with pH$_{T}$^42,47^. To avoid the effect of site-specific correlations between ambient data and pH$_{T}$ time series, the model will be trained independently with the dataset of each location. In this way a proper model calibration is ensured and the prediction power of the model is enhanced. In the Bay of Palma, the model will be used to reconstruct the pH$_{T}$ time series from 2012, exclusively from the points for which the full set of ambient time series data are available (Fig. 1d). This is not possible in Cabrera, due to the fact that no temperature, salinity and DO concentration is available before 2019. Fortunately, these time series do not have the same gaps that the pH$_{T}$ time series exhibits. Thus, we will use the model to fill the gaps in the pH$_{T}$ time series from 2019 to present, as shown in (Fig. 1d).

**Figure 2:**
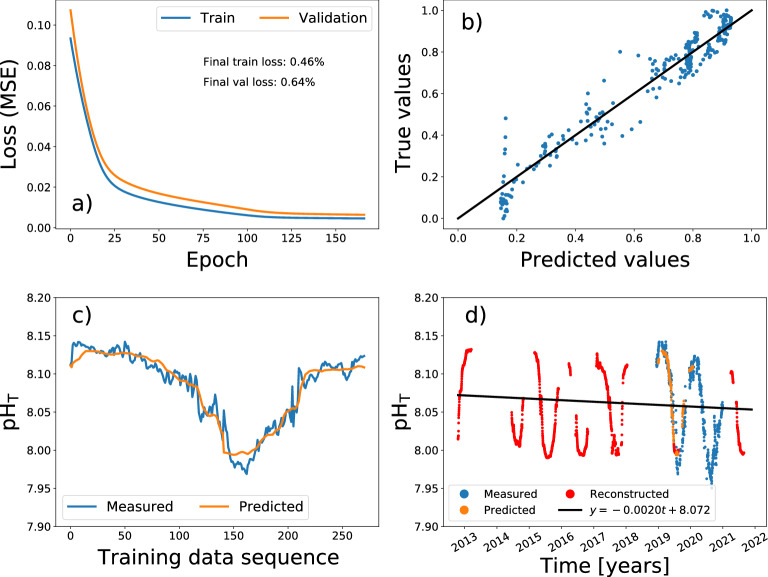
Bidirectional LSTM neural network model applied to assess the decadal pH$_{T}$ trend in the Bay of Palma: (**a**) training process monitoring loss for both training and validation sets, (**b**) predicted pH$_{T}$ values against their true values where the black line is the reference for a perfect prediction, (**c**) predicted pH$_{T}$ time series in the training process (orange) and ground truth series (blue) and (**d**) final prediction for the decadal pH$_{T}$ time series using the output data of the trained model and the measured data. Measured pH data shown in blue, predicted data in the training process is shown in orange and reconstructed data is shown in red. The black line represents the decadal pH trend.

A BiDireccional Long Short-Term Memory (BD-LSTM) neural network (Fig.  S4) was selected as the best recurrent neural network architecture to reconstruct the pH$_{T}$ time series in the Bay of Palma. The training process was successfully completed with no signs of overfitting achieving less than 1% error in both training and validation sets (Fig. 2a). The BD-LSTM neural network was able to fairly predict the majority of the individual pH data points in the time series, although there are some deviations (Fig. 2b). Furthermore, the time series pattern is perfectly captured by the neural network (Fig. 2c). Notice the gaps in the reconstructed pH points (in red) in (Fig. 2d), that are those for which the full ambient time-series is not available. Finally, the reconstructed pH data using the BD-LSTM model was used to assess the decadal trend of acidification in Palma Bay, which yielded $-0.0020$ units of pH per year (black line in Fig. 2d). Indeed, to further characterize this decadal trend, 1000 independent training-prediction processes were carried out using a BD-LSTM neural network. The results showed a mean slope of $-0.0020 \pm 0.00054$ for the decadal acidification trend (see “Methods”).

**Figure 3:**
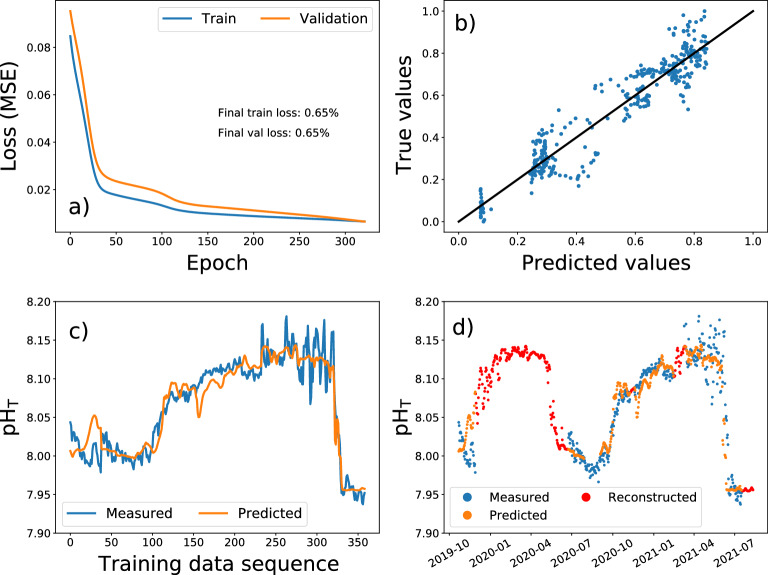
Bidirectional LSTM Neural Network model applied to fill the gaps in the pH$_{T}$ time series in Cabrera: (**a**) training process monitoring loss for both training and validation sets, (**b**) Predicted pH$_{T}$ values against their true values where the black line is the reference for perfect prediction, (**c**) Predicted pH$_{T}$ time series in the training process (orange) and ground truth series (blue) and (**d**) Gaps in the pH$_{T}$ time series filled with the trained model (red), while measured pH$_{T}$ are shown in blue and predicted data in the training process shown in orange.

Regarding the Cabrera data set, with the available ambient time series it is only possible to fill the data gaps, task for which a BD-LSTM neural network was also used. As for the Bay of Palma the training process was successfully completed with no signs of overfitting, yielding less than 1% error in both training and validation dataset (Fig. 3a). The model fairly predicts most of the individual pH$_{T}$ data points in the training dataset, showing some deviations as usual (Fig. 3b). The tendency of the time series is perfectly captured by the model (Fig. 3c) and thus the gap can be filled with reliable data, red points in (Fig. 3d).

## Discussion

The achievement of long term oceanographic data series suitable to evaluate the effects of climate change constitutes a great operational effort which is unequivocally accompanied by partial data loss due to multiple factors (human and instrumental). The advances in the development of pH sensors are enabling the acquisition of precise pH data without identified drift through highly accurate indicator-based spectrophotometric methods^49^. However, in order to determine OA trends, several years of quality seawater pH data are needed, adding more difficulty to the vicissitudes inherent to field work. Recently, the application of computational methods based on Deep Learning (DL) is becoming a useful tool to fill the gaps due to data loss. Several studies have implemented the DL methodology and successfully predicted bio-optical and biogeochemical parameters^44,46–48,50–54^.

Here, the application of a BiDireccional Long Short-Term Memory (BD-LSTM) neural network to predict pH$_{T}$ from physical data, namely temperature, salinity, and dissolved oxygen, the latter as a key indicator of biological activity, permitted the reconstruction of gaps in the time series of pH$_{T}$ and allowed the reconstruction of nine years of pH$_{T}$ data. The BD-LSTM architecture has been proved extremely effective in predicting sequence data, such as time series, as they combine the information for both front and back directions of time (Fig.  S4)^55^, and is more effective (accurate and stable) compared to unidirectional Long Short-Term Memory neural networks. Therefore, in this study the BD-LSTM offered better estimation results over the other neural networks considered to reconstruct time series but also in the completion of missing data.

In the Cabrera station, the BD-LSTM permitted a reliable reconstruction of the gaps in pH$_{T}$ data from December 2019 to June 2020 (Fig. 1d), constituting an advantageous methodology to support the acquisition of long time series data without losing accuracy, as the model can reproduce pH$_{T}$ data with an error lower than 1% (Fig. 3b), closely following the annual variability of the observations (Fig. 3d).

The ability of the BD-LSTM to reconstruct time series was observed through the reconstruction of nine years of pH$_{T}$ data in the Bay of Palma station (Fig. 2d). The modeled pH$_{T}$ data combined with the observations allowed the accomplishment of a long pH time series in order to estimate a pH trend, seasonally adjusted through a sinusoidal fitting, with a rate of decrease of $0.0020\pm 0.00054$ pH units per year ($R^2=0.1$, *p*-value $<0.001$, Fig.  S1), and represents the first estimate of pH trend obtained in the Balearic coastal Sea. Additionally, we applied a linear fit on the reconstructed pH time series obtaining trend of $-0.0025\pm 0.00053$ year$^{-1}$ ($R^2=0.01$, *p*-value $<0.001$). This fit was discarded, because it was shown to introduce a bias in the pH decrease trend.

The observed pH decrease in the Balearic Sea coastal area is well aligned with OA trends reported for open ocean areas, from − 0.0013 pH units year$^{-1}$ in the Munida station (New Zealand) to the high trend found in the Cariaco Basin station up to − 0.0026 pH units per year^27^. The processes associated with the increased pH decline in the Cariaco Basin where related to the upwelling of Subtropical Underwater, rich in dissolved inorganic carbon, thus lowering the pH.

In the Mediterranean Sea, previous annual estimates in open ocean areas ranged from − 0.003 to − 0.0044^19,22^, reflecting the effect of the hydrodynamical and biogeochemical characteristics of the basin on the seawater pH variability^18,56,57^. However, it can be assumed that differences in physical oceanography and ecological processes between areas may modulate local changes of pH. In a coastal Mediterranean area located in the northwestern basin, close to Villefranche-sur-Mer (France), a rate of pH change of $-0.0028\pm 0.0003$ pH units year$^{-1}$ was observed^21^ and attributed principally to atmospheric forcing and secondly to increased warming. The calculated trend of pH decrease due to the atmospheric CO$_{2}$ growth during the period of this study, from 2013 to 2021, was of $0.0025\pm 0.0002$ pH units per year (R$^{2}=0.95$, *p*-value < 0.001), consistently related to the seawater pH decline. Therefore, these analyses suggest that the atmospheric forcing is the main driver responsible for the pH decreasing trend found in the surface coastal Balearic Sea. Subsequently, the difference between the seawater pH decreasing trend obtained and the pH trend calculated from the atmospheric levels could be related to natural biogeochemical processes, not distinctly quantifiable with the available length of the Bay of Palma pH time series.

In addition, the effect of temperature on surface ocean pH, occurring directly through the temperature dependence of the seawater CO$_{2}$ chemistry, as changes in temperature and salinity influence the equilibrium constants of the oceanic CO$_{2}$ system and indirectly through air-sea exchange of CO$_{2}$, can be considered. The influences of these two temperature processes on surface ocean pH has been found responsible of a 50% of the increase in [H$^{+}$] ions, thus a pH decrease, in the surface layers of the Iceland and Irminger Seas^58^. In the Mediterranean Sea northwestern basin, a temperature increase of $0.072\pm 0.022$ $^{\circ }$C year$^{-1}$ was estimated to be responsible for a 40% of the pH decrease^21^. The obtained temperature variability in the Balearic Sea coastal area during this study was of $0.035\pm 0.008$ ($R^2=0.008$, *p*-value $<0.001$, Fig.  S2), indicating that temperature-driven changes could also be assumed to affect the pH trend.

The observed seasonal variability of the data, presented a pH$_{T}$ increase from 7.91 during summer up to 8.18 pH units (Fig. 1d) in winter seasons, clearly followed by the TA values (Fig.  S3). Seasonal changes in TA levels in the study area are ranging from around 2350 to 2550 ${\upmu \text {mol kg}^{-1}}$ (Fig.  S3), largely overtaking the seasonal differences reported previously in the Balearic Sea of up to 50 ${\upmu \text {mol kg}^{-1}}$ in total^59^. This discrepancy in variability could be explained by the intense metabolic processes at the coastal location of the Bay of Palma station. This shallow area has a strong coverage of *Posidonia oceanica*, which due to its high ecosystem production^60^ could be triggering an increase of pH and TA levels, as seen in salinity normalized TA values (NTA, not shown) during winter-spring, due to the uptake of nitrate and phosphate and the calcium carbonate dissolution^59,61^, and during summer, related to the lower community production^62^ a NTA-pH decrease^59^.

Another result from this study worth to mentioning is the obtained decreasing TA trend in the Bay of Palma of − 4.0 ± 0.4 ${\upmu \text {mol kg}^{-1}}$ per year. Although the Western Mediterranean is characterized with lower total alkalinity values in relation to the rest of the basin, resulted from the nearby influence of Atlantic waters, less salty with low-alkalinity water^63,64^, was not expected to influence decreasing decadal TA values. In the northwestern basin, TA values increased over time at a rate of 2.08 ± 0.19 ${\upmu \text {mol kg}^{-1}}$ year$^{-1}$. In the Balearic Sea, the decreasing TA confirm the Atlantic forcing on the alkalinity values and the negligible TA discharges due to rivers in the Balearic Islands. There is a marked south-to-north surface gradient in the western region coupled with the west-to-east gradient of alkalinity in the Mediterranean Sea related to the Atlantic influence^59,65^. Due to a well-established linear relationship of TA and salinity^66^ and the calculated origin of our values^65^ we cannot neglect the strong TA related to the salinity decrease in the study area of − 0.059 ± 0.002 psu per year (R$^{2}=0.25$, *p*-value < 0.001). This rate is in agreement with the salinity decrease found at the coastal site at Villefranche-sur-Mer (− 0.0017 ± 0.0044 psu year$^{-1}$)^21^. Notwithstanding, the intense salinity decrease observed in the Bay of Palma can be linked to a decrease of the intensity of the southern spreading of the Balearic Current trough the Ibiza channel (located between Ibiza and Mallorca Islands) driven by mesoscale processes, and the prevalence of new Atlantic Water coming from the Strait of Gibraltar^67^. Although, this observation is out of the scope of this study and therefore further investigation is needed.

In summary, this work pointed out the useful use of DL techniques, specifically the BD-LSTM architecture, to reconstruct pH data relevant to evaluate seasonal pH variability and to elucidate the climate change consequences, as the OA effect, in a coastal area of the Balearic Sea, which can be extended to the coastal areas of the Western Mediterranean Sea Basin. Nevertheless, future research is necessary to assess and confirm these regional trends, which highlights the importance of maintaining the time series monitoring networks whose data are the base of this study.

## Methods

### Study area

**Figure 4:**
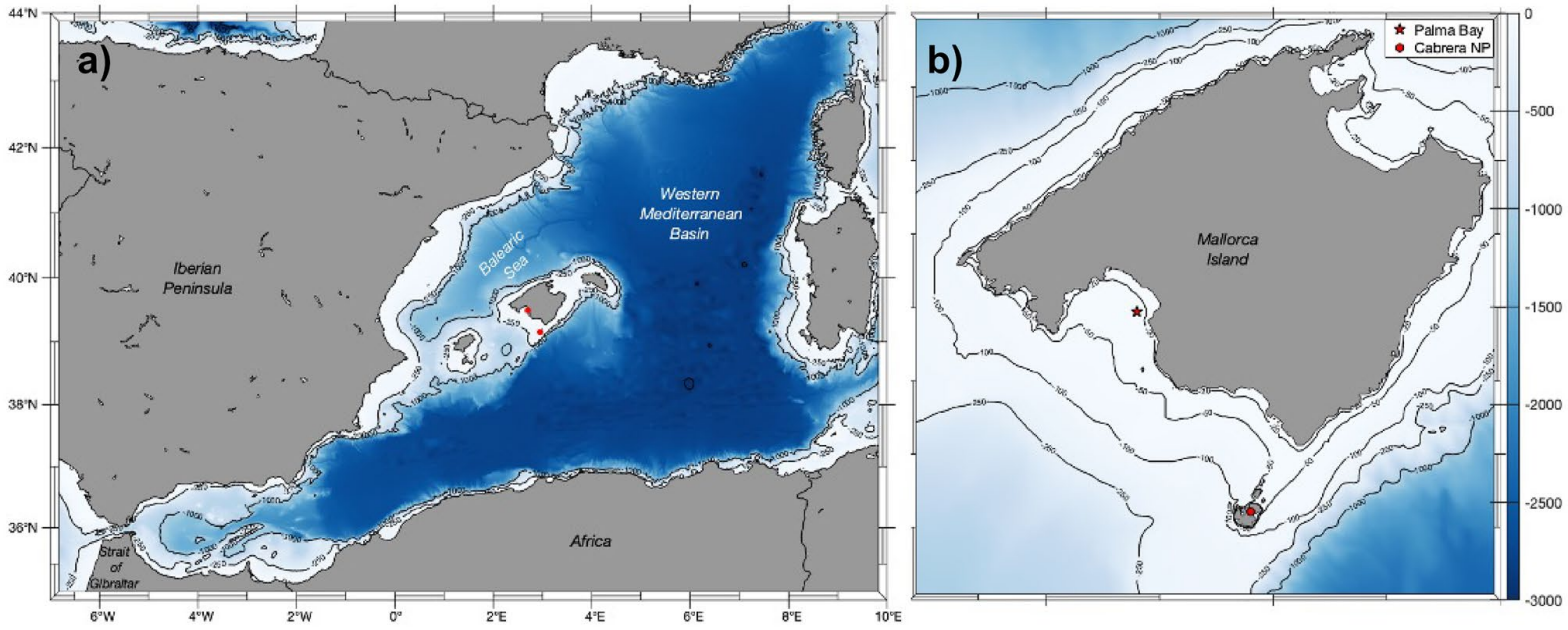
(**a**) Map of the stations location in the Western Mediterranean Sea Basin (red dots) and (**b**) detailed location of the Bay of Palma (red star) and the Cabrera National Park (Cabrera NP, red dot) study sites. Maps were developed with the MATLAB® R2010b software (https://mathworks.com) by using the M_Map toolbox^70^.

We monitored two coastal stations located in the Archipelago of the Balearic Islands in the Western Mediterranean Sea (Fig. 4). One site was positioned within the Bay of Palma (39$^{\circ }$ 29.57088$^{\prime }$ N; 2$^{\circ }$ 42.02430$^{\prime }$ E (Fig. 4b) at a fixed station consisting of an oceanographic buoy managed by the Balearic Islands Coastal Ocean Observing and Forecasting System (SOCIB; https://www.socib.eu/). Here meteorological, hydrological and hydrodynamic data are collected with an hourly frequency since October 2012. The buoy is located at the surface over 20m bottom depth. The Bay of Palma is a large bay with a surface area of 217 km$^{2}$ and approximately 30% seagrass cover^68^. The second station was located at 4 m depth on a mooring line over 8 m bottom depth deployed in the Marine and Terrestrial National Park of the Archipelago of Cabrera (39$^{\circ }$ 9.08217$^{\prime }$ N; 2$^{\circ }$ 57.04767$^{\prime }$ E; Fig. 4b). The mooring line is in a small bay of just under 1 km$^{2}$ and full protection with the largest meadow of the archipelago, covering 89.1% of the surface area between 0 and 10 m depth^69^. Neither site has important freshwater inputs. Both stations are part of the Balearic Ocean Acidification Time Series (BOATS) network included in the Interdisciplinary Thematic Platform: Water:iOS (https://pti-waterios.csic.es/).

### Data collection

In both stations a SAMI-pH (Sunburst Sensors LCC) was attached, at 1 m in the Bay of Palma and at 4 m depth in Cabrera. The pH sensors were measuring pH, in the total scale (pH$_{T}$), hourly since December 2018 in the Bay of Palma and since November 2019 in Cabrera. The sensor precision and accuracy are < 0.001 pH and ± 0.003 pH units, respectively. Monthly maintenance of the sensors was performed including data download and surface cleaning.

Temperature and salinity from the Bay of Palma oceanographic buoy was obtained from October 2012 and for the Cabrera mooring line from November 2019 with a CT SBE37 (Sea-Bird Scientific©) in both stations. Accuracy of the CT is ± 0.002 $^{\circ }$C for temperature and ± 0.003 mS cm$^{-1}$ for conductivity. Additionally, oxygen data from a SBE 63 (Sea-Bird Scientific ©) sensor attached to the CT in Cabrera and from a YSI 6600V2-4 Multiparameter Water Quality Sonde with a 6450 ROX DO sensor (Yellow Spring Instruments Inc. ©)^71^ and a miniDot (PME, Inc. ©) in the Bay of Palma were used. Accuracy of oxygen sensors is ± 2%, ± 1% and ± 5% for the SBE 63, the YSI and the miniDot, respectively.

Periodically water samplings for dissolved oxygen (DO), pH in total scale at 25 $^{\circ }$C (pH$_{T25}$) and total alkalinity (TA) were obtained during the sensor maintenance campaigns. DO and (pH$_{T25}$) samples were collected in order to validate the data obtained by the sensors.

DO concentrations were evaluated with the Winkler method modified by Benson and Krause^72^ by potentiometric titration with a Metrohm 808 Titrando with a accuracy of the method of ± 2.9 ${\upmu \text {mol kg}^{-1}}$ and with an obtained standard deviation from the sensors data and the water samples collected of ± 5.9 ${\upmu \text {mol kg}^{-1}}$.

pH$_{T25}$ data was obtained by the spectrophotometric method with a Shimadzu UV-2501 spectrophotometer containing a 25 $^{\circ }$C-thermostated cells with unpurified *m*-cresol purple as indicator following the methodology established by Clayton and Byrne^73^ by using Certified Reference Material (CRM Batch #176 supplied by Prof. Andrew Dickson, Scripps Institution of Oceanography, La Jolla, CA, USA). The accuracy obtained from the CRM Batch was of ± 0.0051 pH units and the precision of the method of ± 0.0034 pH units. The mean difference between the SAMI-pH and discrete samples was of 0.0017 pH units.

TA samples were collected in 50 ml Falcom vials and poisoned with 20 ${\upmu }$L of HgCl$_{2}$ and determined by open cell potentiometric titration with a Titrando 808 (Metrohm) following the Standard Operation Procedure (SOP) 3b^74^. TA values were also calculated from the temperature and salinity values obtained in the Bay of Palma from 2012 by using a second-order polynomial model for TA specifically described for the Mediterranean Basin^65^.

pH values due to the atmospheric CO$_{2}$ levels were estimated by using the CO2SYSv3 program^75^, with the most internally consistent and preferred carbon^76,77^ and sulphate dissociation constants^78^ for current surface ocean studies^79^, with the Bay of Palma in situ temperature and salinity, the calculated TA values and the atmospheric CO$_{2}$ levels converted from dry air to wet^80^ as inputs. Carbon dioxide (CO$_{2}$) atmospheric molar fraction used was obtained from the monitoring station of Lampedusa (LMP), Italy of the NOAA (National Oceanic and Atmospheric Administration, USA) monitoring network^81^.

### Data processing

Once data was validated, several processing steps were performed to ensure an optimal training process for the neural network models. First, all the data of the time series were re-sampled by averaging the data points obtaining a daily frequency. Afterwards, a standard feature-scaling procedure (min-max normalization) was applied to every feature (temperature, salinity and oxygen) and to pH$_{T}$. Finally, we built our training and validations sets as tensors with dimensions $(\text {batch}_\text {size}, \text {window}_\text {size}, N_{\text {features}})$, where $\text {batch}_\text {size}$ is the number of examples to train per iteration, $\text {window}_\text {size}$ is the number of past and future points considered and $N_{\text {features}}$ is the number of features used to predict the target series. Temperature values below $T=12.5$ °C were discarded as they are considered outliers in sensor data outside the normal range in the study area.

### Computing the trend of seasonal data

The trend of seasonal time-series is often computed by means of statistical methods based on moving averages or more advanced techniques such as the Seasonal Trend Decomposition Loess^82^. Nevertheless, these procedures do not work with gappy time series, so that a different approach is needed. In this work we fitted the following oscillatory function with trend to our data:

1 $$\begin{aligned} y(t)=A\sin (\omega t+\phi )+Bt+C \ , \end{aligned}$$

where the parameter *B* corresponds to the trend of the data.

Moreover, after this fit, the seasonal component ($A\sin (\omega t + \phi )$) can be removed from the original time-series and a standard linear regression can be performed to the transformed data to obtain the trend (which is exactly *B*) with the $R^2$ and *p*-value estimates given by the linear regression (Figs.  S1,  S2).

### Selecting the best neural network architecture

Several recurrent neural network (RNN) architectures were considered as candidates to reconstruct the pH time series, including a Simple Recurrent neural network (SRNN), Long-Short Term Memory (LSTM), BiDirectional LSTM (BD-LSTM, (Fig.  S4) and BiDirectional Gated Recurrent Unit (BD-GRU).

Initially, manual tests were performed on each architecture to determine the optimal set of parameters that yielded the best possible results. These tests were based on minimizing the errors in both training and validation set while avoiding overfitting. To avoid overfitting, we implemented automated callbacks to stop the training process whenever the validation loss increased or crossed the training loss. During this test we determined the minimum number of nodes, which helps in avoiding overfitting and the minimum window size, which allows to use the most possible number of data points for training and prediction. All the RNNs were trained in batches of size 32. To enhance clarity and accessibility, the optimal values obtained for the more relevant parameters are summarized in Table 1.

**Table 1:** Optimal parameters used for the different RNN architectures.

	Hidden layers	Nodes/cells	Window size	Activation function	Output function	Loss	Learning rate	Optimizer
SRNN	1	3	6	Tanh	Sigmoid	MSE	0.01	Adam
LSTM	1	3	6	Tanh	Sigmoid	MSE	0.01	Adam
BD-LSTM	1	3	6	Tanh	Sigmoid	MSE	0.01	Adam
BD-GRU	1	1	6	Tanh	Sigmoid	MSE	0.01	Adam

In order to identify the best-performing architecture an automated procedure was developed to statistically compare the outputs of each model. Each architecture was trained in 1000 independent processes, ensuring a final training mean-squared error of less than $0.8\%$ while avoiding overfitting implementing the previously mentioned callbacks. The code used for the analysis can be found in^83^.

In Table 2, a summary of the statistical results obtained for each architecture is presented. All architectures provide similar training and validation errors and provide similar results for the decadal pH$_{T}$ trend, predicting a slope of around $-0.0020$ pH units per year with an intercept of 8.07 pH units. However, the BD-LSTM turns out to be the architecture providing most accurate (smallest training and validation errors) and precise (smallest statistical error) results (Fig.  S4). Thus, we selected the BD-LSTM neural network as the best architecture to reconstruct the pH$_{T}$ time series. Data corresponding to the Bay of Palma were used in the selection of the best neural network architecture.

**Table 2:** Statistical comparison between different RNN architectures.

	Slope	Intercept	Training error	Validation error	Training epochs	Training time
RNN	− 0.0021 ± 0.00077	8.07 ± 0.006	0.54 ± 0.08	0.72 ± 0.12	293 ± 95	15.52 ± 4.75
LSTM	− 0.0018 ± 0.00067	8.06 ± 0.005	0.49 ± 0.03	0.68 ± 0.05	245 ± 68	17.55 ± 4.21
BD-LSTM	− 0.0020 ± 0.00054	8.07 ± 0.004	0.46 ± 0.03	0.64 ± 0.04	167 ± 45	15.13 ± 3.00
BD-GRU	− 0.0020 ± 0.00066	8.07 ± 0.005	0.51 ± 0.07	0.74 ± 0.10	347 ± 95	27.68 ± 6.84

The code and data used to determine the best neural network architecture can be found in a GitHub repository^83^.

## Supplementary Information

Supplementary Information.
