# Supplementary material for: pH trends and seasonal cycle in the coastal Balearic Sea reconstructed through machine learning
Source: Sci Rep. 2022 Jul 28;12:12956. doi: 10.1038/s41598-022-17253-5 (PMC9333055; doi:10.1038/s41598-022-17253-5)
Supplement: Supplementary file 1 — Supplementary Information. [file 41598_2022_17253_MOESM1_ESM.pdf]

Supplementary Information

Seasonal adjusted fits for pH and temperature

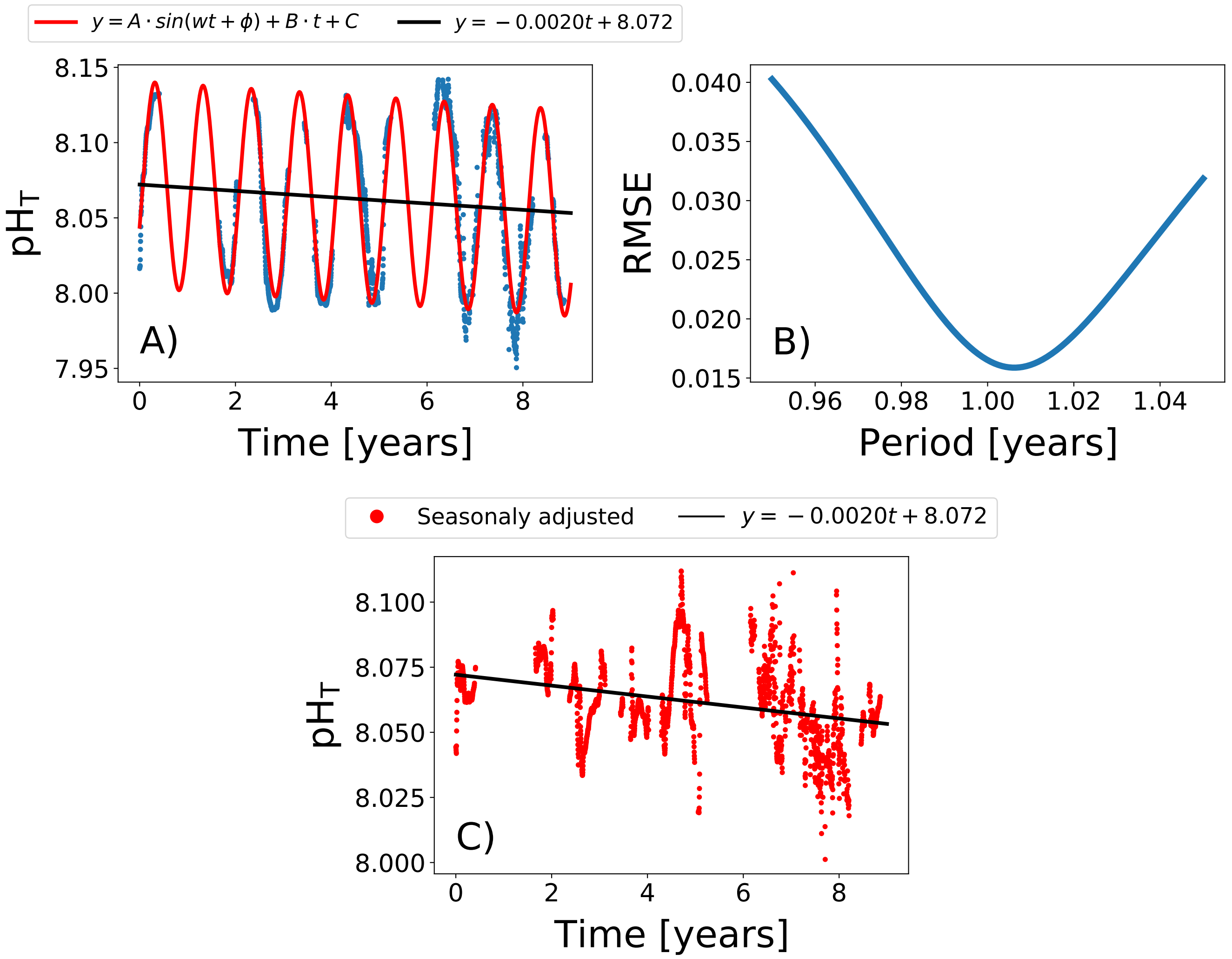

Figure S1. Seasonally adjusted fit to reconstructed and measured pH data. A) Full fit of Eq. (1) with $A = 0.0686 \pm 0.0005$, $B = -0.0020 \pm 0.0002$, $\phi = -6.704 \pm 0.008$, $C = 8.0721 \pm 0.0008$. B) Optimal period ($T = 1.006$, $\omega = 2\pi/T$) found to fit the data. C) Linear regression to the seasonal adjusted data.

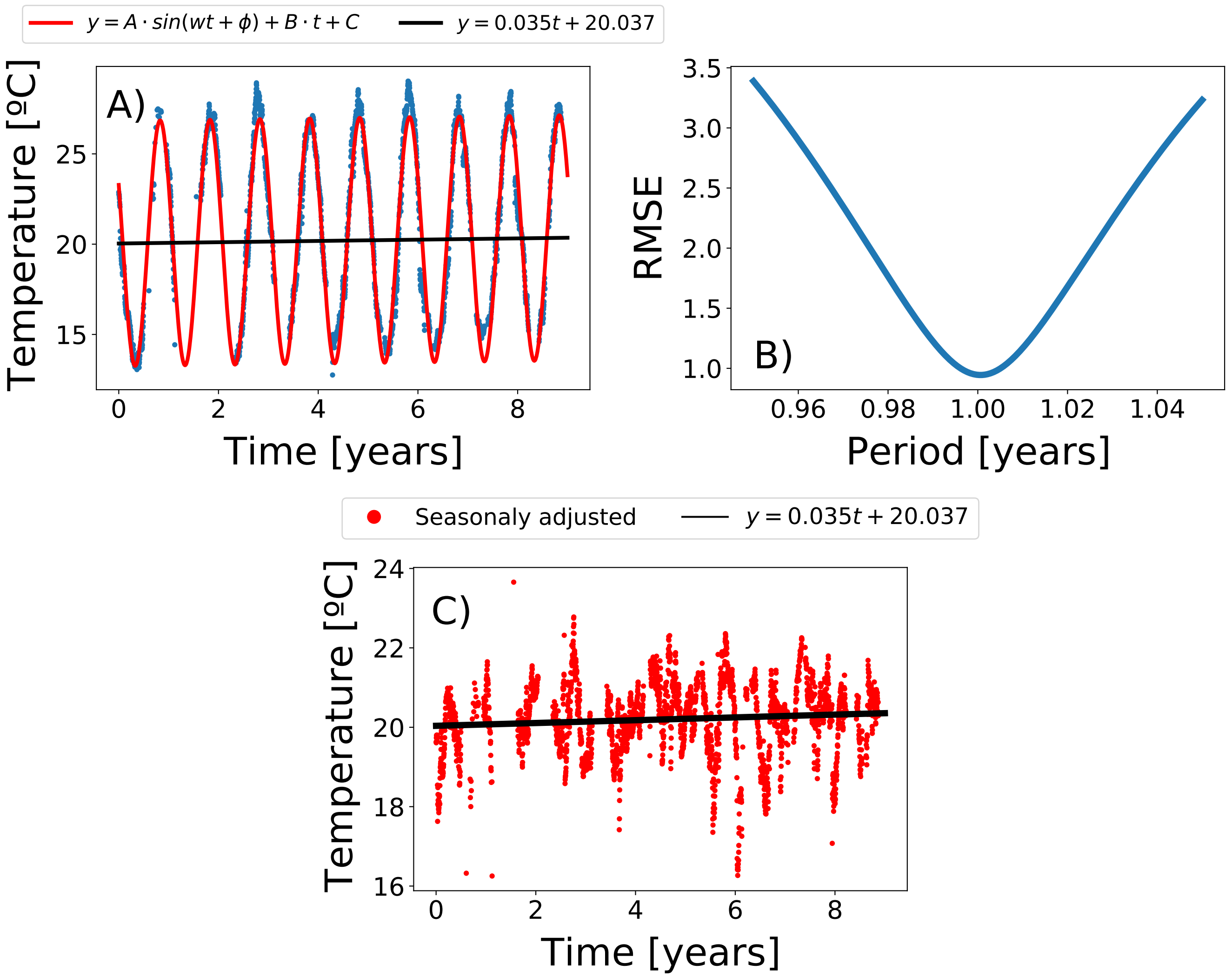

Figure S2. Seasonally adjusted fit to reconstructed and measured temperature data. A) Full fit of Eq. (1) with $A = 6.792 \pm 0.029$, $B = -0.0353 \pm 0.0080$, $\phi = -3.6396 \pm 0.0040$, $C = 20.037 \pm 0.043$. B) Optimal period ($T = 1.0$, $\omega = 2\pi/T$) found to fit the data. C) Linear regression to the seasonal adjusted data.

Total alkalinity in the Bay of Palma

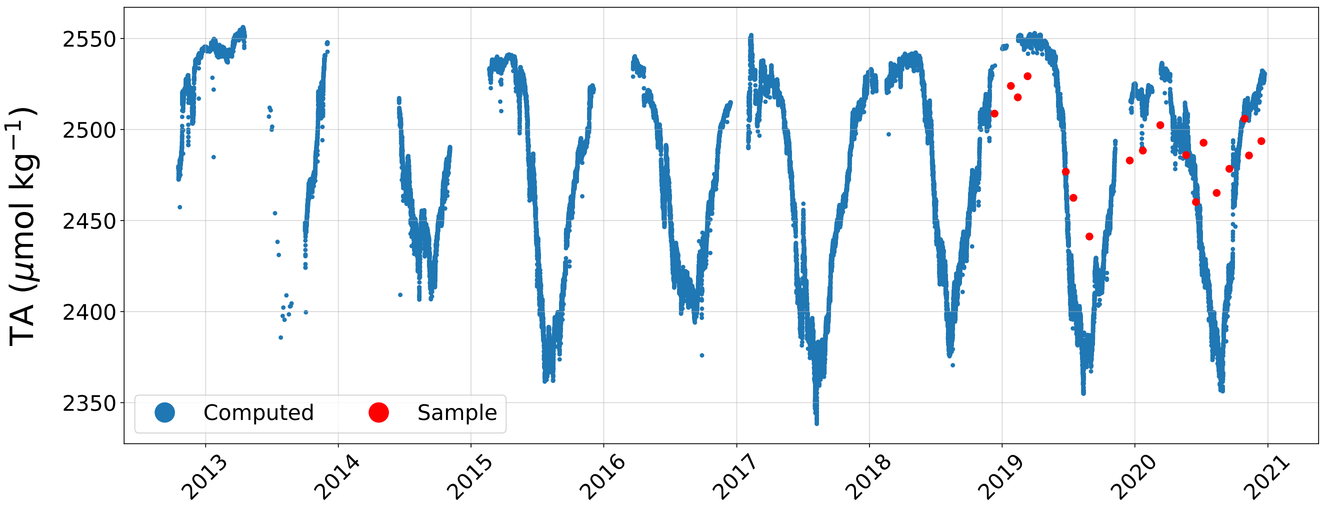

Figure S3. Total alkalinity in μmol kg^{-1} calculated values (blue dots) and concentrations obtained from samples (red dots) in the Bay of Palma.

Bidirectional Long-Short Term Memory neural network

Recurrent Neural Networks (RNNs) are a class of artificial neural networks in which node connections arise along a temporal sequence, i.e. previous values in a time series are linked to current values. In simple words, RNNs predict a point of the time series using past information. Simple Recurrent Neural Networks (SRNNs) are the straightforward extension of Feedforward Neural Networks, in which past information and learned knowledge is encoded in the network as state vectors. SRNNs suffer from the so-called vanishing gradient problem, i.e. distant parts of the time series do not play a role in the training process. Thus, SRNNs are not capable to learn long-term dependencies. Long-Short Term Memory (LSTM) neural networks overcome this limitation by implementing three gates to update and control the cell state (forget gate, input gate, output gate), thus allowing to keep long-term dependencies[?]. Bidirectional Long-Short Term Memory (BD-LSTM) neural networks are able to encode both past and future information by implementing two LSTM layers flowing in opposite time directions. The forward layer preserve past information while the backwards layer preserves future information.Thus, using the two hidden states combined BD-LSTM are able in any point in time to preserve information from both past and future. A schematic representation of the BD-LSTM neural network is shown in (Fig. S4).

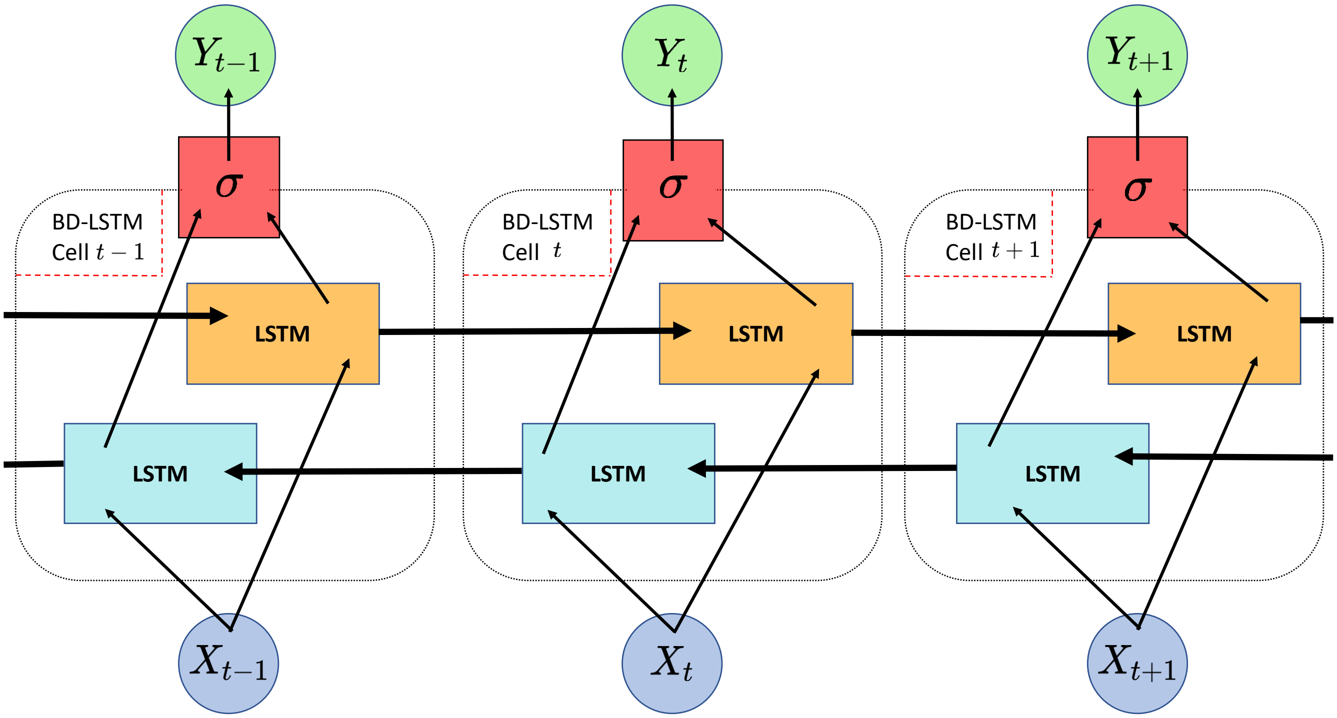

Figure S4. Scheme for the Bidirectional-LSTM Neural Network. The network receives as input a tensor of shape $(\text{batch}_{\text{size}}, \text{window\_size}, N_{\text{features}})$, where $\text{batch}_{\text{size}}$ is the number of examples to train per iteration, $\text{batch}_{\text{size}}$ is the number of past and future points considered and N_{features} is the number of features used to predict the target series. First, the input is linearly transformed to match the number of cells of the network (3 in the figure). Then, the input is transformed by the backward (blue) and forward (orange) LSTM layers, which propagates information backwards and forwards, respectively. Finally, the values are transformed through an activation gate. In our model, this values will be ultimately transformed via a dense layer to a unique output.
